# WeChat-Based Intervention for Chinese Immigrants With Hypertension: Development and Evaluation Study

**DOI:** 10.2196/45769

**Published:** 2023-07-27

**Authors:** Wen-Wen Li, Prisca Toh

**Affiliations:** 1 School of Nursing San Francisco State University San Francisco, CA United States

**Keywords:** social media, hypertension, medication adherence, Chinese immigrants, WeChat, blood pressure, BP

## Abstract

**Background:**

Despite Chinese immigrants having a higher or comparable proportion of hypertension (HTN) compared to non-Hispanic White and Hispanic individuals, there are no effective technology-based intervention studies that target HTN management in Chinese immigrants in the United States.

**Objective:**

The aim of this study was to develop and pilot-test the efficacy of a culturally and linguistically sensitive social media–based intervention (WeChat) for Chinese immigrants to improve blood pressure (BP) control, antihypertensive medication adherence, and self-efficacy.

**Methods:**

The study was conducted in 2020 with a pre- and posttest design (N=20). A WeChat-based intervention was implemented using one 20-minute video presentation plus one 20-minute nurse counseling session via WeChat at the baseline, followed by 4 biweekly 20-minute nurse counseling sessions via WeChat calls. The primary outcome (BP control) and secondary outcomes, including medication adherence and self-efficacy in HTN management, were measured at baseline and at 6 months.

**Results:**

The participants’ mean age was 68.9 (SD 10.2; range: 51-86) years. The majority of the participants were female (n=13, 65%), had a high school degree or less (n=15, 75%), were married (n=16, 80%), not religious (n=13, 65%), and not employed (n=12, 60%). The results showed that the mean baseline systolic BP was 131.43 (SD 9.61) mmHg, and the mean diastolic BP was 79.79 (SD 9.62) mmHg. The 6-month outcome showed a reduction of systolic BP (–0.74, SD 9.18 mmHg; *P*=.05) and diastolic BP (–0.96, SD 6.92 mmHg; *P*=.001). The mean score for medication adherence at baseline was 4.50 (SD 1.70), and it significantly improved to 3.65 (SD 1.18) at 6 months (reversely scored; possible range was 1-5, with 1 being the best score; *P*=.001). Self-efficacy in HTN management had a trend in reduction from a baseline score of 8.28 (SD 1.25) decreasing to 7.93 (SD 1.48) at 6 months, with a mean difference of 0.34 (SD 2.02), with a score of 0 indicating the lowest self-efficacy and a score of 10 indicating the highest self-efficacy.

**Conclusions:**

Our WeChat-based HTN management program showed a significant improvement in diastolic BP and medication adherence as well as a trend of reduction for systolic BP and self-efficacy in managing HTN in Chinese immigrants. Compared to the traditional health care system, the proposed WeChat-based HTN management program has a low cost and is easy to implement. Thus, further investigation is recommended to generate further results. This intervention should be tested across different regions and clinical settings to verify the findings. The long-term goal is to implement the intervention in clinical settings to help Chinese immigrants at large achieve better HTN management.

## Introduction

### Overview

The number one cause of premature morbidity and mortality in the United States is hypertension (HTN) [1,2]. Despite Chinese immigrants having a higher or comparable proportion of HTN (40%) compared to non-Hispanic White (38%) and Hispanic (40%) individuals [3-5] and being at high risk for HTN-related health complications, they are underrepresented in health-related research [6]. A recent study has found that technology-based HTN interventions, such as video presentations or home monitors, showed efficacy, were accepted, and had positive behavioral outcomes among Black and Hispanic populations [7,8]. However, despite being a part of the fastest-growing Asian ethnic population, there are no effective intervention studies that target HTN management in Chinese immigrants in the United States [7,8]. Furthermore, the existing technology-based HTN interventions lack culturally sensitive educational materials [7]. For example, due to dietary differences, the usual methods for restricting sodium intake and eating heart-healthy diets do not work well in Chinese immigrants. Instead, studies have found that substituting Chinese herbs or spices to reduce sodium intake may be a more effective intervention [9]. To address the gap in current HTN management interventions, which includes a lack of using technology and culturally sensitive approaches, this study implemented Chinese Medicine as Longevity Modality (CALM); CALM combined both methods, that is, technology-based and culturally sensitive components. The CALM intervention was initially developed by the first author (WWL) in 2015 [9]. In the first stage, an educational video on culturally sensitive management of HTN (eg, the use of Chinese herb and spices to replace excessive sodium intake) was developed. Subsequently, additional components were added in 2020 (for this pilot study), including the use of a storytelling video to exemplify how to culturally manage HTN and the use of a social media platform, WeChat, to interact with patients remotely on HTN management. With this 2-pronged approach, the WeChat-based CALM intervention aimed to improve blood pressure (BP) control (primary outcome) and other secondary outcomes, including medication adherence and self-efficacy. In this paper, the WeChat-based intervention will be used to refer to the WeChat-based CALM intervention.

WeChat is one of the most popular social networking media apps among Chinese immigrants. WeChat, installed on a smart phone, iPad, and computer or laptop, is an affordable method of delivering health-related information to a wide audience of Chinese immigrants. WeChat is one of the few social medial platforms that can be used when patients travel to China. Other popular social media platforms, such as Facebook, Messenger, LINE, and WhatsApp, cannot be used in China. For studies that need to follow up on Chinese participants traveling to China, WeChat is a realistic possibility.

### Aims and Innovation

The specific aim was to test the feasibility and efficacy of the WeChat-based intervention in improving HTN control, medication adherence, and self-efficacy in managing HTN. If the WeChat intervention is found to be effective, it could potentially be adapted to help Chinese immigrants improve their HTN management in broader clinical settings.

## Methods

### Procedure Overview

This study was conducted with a 1-group, pre- and posttest design. Data were collected via self-report questionnaires for demographic information, medication adherence, physical activities, and confidence in following the HTN regimen and salt intake. BP was also measured.

### Ethics Approval

Institutional review board approval was obtained from San Francisco State University (X17-41).

### Setting

Participants were recruited in 2020 from a low-income housing apartment complex for older adults subsidized by the US Housing and Urban Development Division. It is located near Chinatown in the San Francisco Bay Area. It serves approximately 200 older citizens, 80% of whom are Chinese immigrants.

### Sample

A convenience sample of 20 Chinese immigrants with HTN was recruited from the aforementioned housing apartment complex for older adults. Inclusion criteria were the following: (1) self-identified as a Chinese immigrant aged 18 years and older; (2) having a diagnosis of HTN for at least one year; (3) having taken HTN medications for more than 1 month prior to study enrollment; and (4) being able to speak and read Chinese. Exclusion criteria were based on self-report, as follows: being medically unstable or having concurrent psychiatric problems.

### Sample Size

The sample size (N=20) was determined based on the resources available in the study period and the study objectives, which aimed to pilot-test the intervention.

### Measurements

All questionnaires, including demographic information, clinical factors, and medication adherence were administered using pencil and paper and completed by the study participants.

### Descriptive Factors

Demographic factors were modified from the national guideline developed by the Centers for Disease and Control and Prevention [10]. Measurements of language and cultural factors, such as immigrant status and location of birth, were developed from our previous studies [10]. Duration of HTN diagnosis (in years) referred to the duration of time from the first diagnosis of HTN until the study interview [10].

### Primary Outcome

BP measurement was the primary outcome. An Omron brand digital BP machine (code HEM-7201) was used to measure participants’ BP following the standard processes identified by the Joint National Committee VII affiliated with National Heart, Lung, and Blood Institute (). BP measurements were obtained twice. The values for systolic and diastolic BPs were averaged.

### Secondary Outcomes

#### Measurement of Medication Adherence

The Medication Adherence Scale measures adherence to medication with 3 scales—whether patients missed, forgot, or were not careful about taking their medication [10]. A Likert scale ranging from “None of the time” to “All of the time” was used. The Cronbach α was .65 [10]. The total scores for the 3 scales were summed, ranging from 3 to 15. The lowest score (3) represented the best adherence, and the highest score (15) represented the poorest adherence (reverse scored).

#### Measurement of Self-Efficacy in Managing HTN

The Self-Efficacy in Managing Hypertension Scale measures patients’ self-efficacy in managing their HTN through 6 items. A scale ranging from 1 (no self-efficacy) to 10 (highest self-efficacy) was used. The Cronbach α was .91 [10]. The total scores for the 6 items were summed, with a range of 6 to 60. The lowest score (6) represented the lowest efficacy, and the highest score (60) represented the highest efficacy.

### Study Procedures

Before the study launch, 1 bilingual and bicultural intervention registered nurse (RN) was recruited and trained in the study procedures. The RN was able to speak 2 dialects of Chinese, including Mandarin and Cantonese, which were used to interview the study participants. This RN had extensive experience working in hospitals, which included previously working as a nursing assistant and working with Chinese patients. The RN was trained for 2 days by the principal investigator in the following areas: (1) basic HTN information, (2) pharmacological and nonpharmacological HTN treatment, (3) BP measurement following the Joint National Committee VII guideline, (4) interview and counseling techniques, and (5) practicing role playing for interviewing and counseling.

The intervention RN obtained a written consent indicating agreement to participate during the initial visit with the study participants. The participants then filled out self-reported questionnaires for sociodemographic and cultural data as well as a health and smoking history.

Once completed, the questionnaires were reviewed for completion by the RN. The RN then measured the participant’s BP twice in a sitting position. As mentioned earlier, 2 readings were averaged and used as baseline data. If the 2 BP measurements differed by more than 5 mmHg, another BP measurement was taken. The average among the 3 measurements was recorded.

Additionally, participants viewed an educational and storytelling video (20 mins) via YouTube using a laptop provided by the research RN. The video was narrated by 4 Chinese immigrants sharing how they had a stroke because of uncontrolled HTN and how to prevent another episode of stroke by optimizing their BP control via practice using both Western (taking BP medication regularly) and Chinese medicine (eg, acupuncture and doing Tai Chi). The details of video development were presented and published previously [9]. At the end of the video, the RN discussed strategies for HTN management, including common culturally specific barriers or solutions. For instance, if patients perceived herbs to be superior to Western drugs, the RN would discuss with them about why they thought herbs were superior, how to balance the intake of herbs and Western medications, and the importance of adherence to Western drugs. During the discussion, the RN assessed participants’ lifestyles and suggested specific culturally congruent strategies to facilitate BP control via improvement of medication adherence, physical activity, weight change, and sodium intake. There was time for participants to ask questions and request clarification.

Follow-up sessions through both WeChat and in-person office visits were then scheduled by the RN. A total of 4 WeChat phone calls were scheduled for the intervention group at 2, 4, 6, and 8 weeks. These calls discussed issues relating to HTN management, such as medication adherence, physical activity, weight change, and sodium intake. At 3 months, participants came in for an office visit to measure their BP, and the RN assessed if they needed a referral to cardiology if their BP remained high. No intervention was given. Participants received a shopping bag as a token of appreciation for their time and effort for participating in the study. At 6 months, participants came in for a final office visit to conclude the study. The primary outcome (ie, BP control) and secondary outcomes, including medication adherence and self-efficacy in HTN management, were measured at 6 months. No intervention was given. Participants were given US $40 gift cards as an appreciation for their time and effort.

### Data Analysis

All data were analyzed using IBM SPSS Statistics (version 27; IBM Corp). Descriptive statistics were used to screen data for missing values and outliers and to describe the demographic and clinical variables. A paired sample *t* test (2-tailed) was used to examine the difference between pre- and postintervention in terms of change in BP and medication adherence. Statistical significance was set at .05.

## Results

### Sample Characteristics

Table 1 shows study participants’ demographic, cultural, and clinical data. The mean age was 68.9 (SD 10.2; range 51-86) years. The majority of the participants were female (n=13, 65%), had less than (or) a high school degree (n=15, 75%), were married (n=16, 80%), not religious (n=13, 65%), and not employed (n=12, 60%). Participants had the following cultural and linguistic background: first-generation Chinese (n=17, 85%), born in Mainland China (n=17, 85%), and spoke Cantonese (n=15, 75%). The average number of years living in the United States was 20.9 (SD 13.6) years, ranging from 2 to 55 years. The average years of HTN diagnosis was 8.9 (SD 5.5) years, ranging from 3 to 22 years.

**Table 1:** Baseline characteristics of Chinese immigrants with hypertension (N=20).

Variables											Values
Age (years), mean (SD; range)											68.7 (10.2; 51-86)
**Gender, n (%)**											
	Women									13 (65)	
	Men									7 (35)	
**Education, n (%)**											
		Primary school									4 (20)
		Middle school									5 (25)
		High school									6 (30)
		Associate’s degree									3 (15)
		Bachelor’s degree									1 (5)
		Master’s degree and above									1 (5)
**Marital status, n (%)**											
			Married							16 (80)	
			Divorced or separated							1 (5)	
			Single							1 (5)	
			Widowed							1 (5)	
			Missing							1 (5)	
**Religion, n (%)**											
				None						13 (65)	
				Buddhism						3 (15)	
				Catholic						1 (5)	
				Christian						2 (10)	
				Missing						1 (5)	
**Are you living with your family or friends(s)? n (%)**											
					No					1 (5)	
					Yes					18 (90)	
					Missing					1 (5)	
**Employment, n (%)**											
						Retired				7 (35)	
						Part-time				1 (5)	
						Not employed				12 (60)	
**Generation, n (%)**											
						First generation				17 (85)	
						Second generation				3 (15)	
**Location of birth, n (%)**											
							Mainland China				15 (75)
							Taiwan				2 (10)
							Hong Kong				2 (10)
							Other				1 (5)
**Annual income (US $), n (%)**											
								≤9999		12 (60)	
								10,000-99,999		3 (15)	
								20,000-29,999		1 (5)	
								50,000-59,999		1 (5)	
								Refused to answer		3 (15)	
**Language used to communicate with doctor or nurse, n (%)**											
									Cantonese	15 (75)	
									Mandarin	5 (25)	
Baseline BP^a^ control rate (systolic BP<130 mmHg and diastolic BP<80 mmHg)											2 (10)

^a^BP: blood pressure.

In terms of a response rate for the study participation, 29 participants were approached, and 20 were enrolled in the study, resulting in 69% (20/29) response rate. For those 9 participants who refused to take part in the study, the reasons were as follows: could not read Chinese (n=1); were not interested in the study (n=5); did not want to provide personal information, such as demographics (n=1); and were too busy to participate in several sessions of the interview process (n=2).

### Change in Outcomes Over a 2-Month Period

Table 2 represents the BP changes over the duration of the study.

Table 2 shows that the mean baseline systolic BP was 131.43 (SD 9.61) mmHg, and the mean diastolic BP was 79.79 (SD 9.62) mmHg. The 6-month outcome showed a reduction of systolic BP (–0.74, SD 9.18 mmHg; *P*=.05) and diastolic BP (–0.96, SD 6.92 mmHg; *P*=.001). The mean score for medication adherence at baseline was 4.50 (SD 1.70), and it significantly improved to 3.65 (SD 1.18) at 6 months (reverse scored, with a possible range being 1-5 and 1 indicating the best score). Self-efficacy in HTN management had a trend in reduction from a baseline of 8.28 (SD 1.25) to 7.93 (SD 1.48) at 6 months, with a mean difference of 0.34 (SD 2.02). In this scale, a score of 0 represents the least efficacious, and a score of 10 indicates the most efficacious.

**Table 2:** Changes in outcomes over the 6-month period. Italicized *P* values are statistically significant.

Outcomes	Outcome change			
	Baseline, mean (SD)	6 months, mean (SD)	Mean difference (SD)	*P* value
Systolic blood pressure	131.43 (9.61)	130.68 (7.37)	–0.74 (9.18)	*.05*
Diastolic blood pressure	79.79 (9.62)	78.83 (7.94)	–0.96 (6.92)	*.001*
Medication adherence (reservedly scored)	4.50 (1.70)	3.65 (1.18)	0.85 (0.90)	*.001*
Self-efficacy in managing hypertension	8.28 (1.25)	7.93 (1.48)	0.34 (2.02)	.74

## Discussion

### Principal Findings

This study tested the efficacy of a culturally and linguistically sensitive social media–based intervention (WeChat) in Chinese immigrants with HTN to evaluate its efficacy in improving BP control and medication adherence. In a sample of 20 participants, the majority were first-generation, Cantonese-speaking female individuals born in mainland China.

The results revealed that our social media–based intervention led to a significant improvement in the primary outcomes, including systolic and diastolic BP, as well as the secondary outcome of medication adherence. There was no significant difference for the other secondary outcome, which was self-efficacy.

### Comparison With Prior Work

The reduction in BP was significant but on a small scale both in our study and in the study by Bray et al [11] (–0.74 vs –5.4 mmHg for systolic BP and –0.96 vs –2.7 mmHg for diastolic BP). The consistent results may be due to similarities between the 2 interventions (ie, our WeChat-based intervention and Bray et al’s home BP intervention) and due to intervention simplicity and intensity.

Regarding intervention simplicity, we asked the participants to watch a video (20 mins) and engage in counseling (20 mins) with a nurse to individualize their self-management of HTN. The subsequent WeChat calls (5 mins/time) were simply to follow up with each participant to discuss any issues and solutions for their individualized self-management of HTN. Bray et al [11] asked participants to take an initial training session lasting 40-50 minutes on how to perform home BP monitoring, to transfer data electronically to the research office, and self-titrate BP medications. Accordingly, the research team followed up with each participant once per month for safety advice in the case of high and low BP readings via phone calls.

In terms of intervention intensity, our WeChat study had participants watch one 20-minute video plus a 20-minute counselling session for the initial visit followed by 4 WeChat calls (5 min/time). The total intervention time was 60 minutes. Bray et al [11] also implemented the initial visit for 40-50 minutes followed by a monthly phone call interaction for 12 months (estimated total intervention time: 100-110 mins).

In taking a detailed look, Bray et al’s [11] participants showed slightly greater reductions in both systolic and diastolic BP compared to our study participants, which may be explained by the following factors: first, the longer duration of the study and the larger sample size could both contribute to a more accurate observation of BP changes. Bray et al [11] had a sample of 263 participants, and the study was conducted over 12 months. A systematic review on a randomized controlled trial for Asian American lifestyle interventions [8] also showed that half of the studies had a sample size of fewer than 100 subjects, which hindered the assessment of intervention effectiveness. Thus, our smaller sample size with a shorter period may hinder the efficacy of our WeChat-based intervention. Second, the higher baseline BP readings may exhibit greater improvement of BP that may be observed. For instance, Bray et al [11] recruited participants with a systolic BP140 mmHg, while our study’s average baseline systolic BP was 131.4 (SD 9.61) mmHg. The higher levels of baseline systolic BP [11] may explain why they observed more BP improvement in their study compared to our study.

Bove et al [12] had a larger-scale reduction in systolic BP (–18.2 mmHg). This study was conducted over a period of 4 years, with a sample of 241 participants. In addition, Bove et al [12] required patients to have a systolic BP 140 mmHg, the same requirement as Bray et al [11]. Our study did not have a requirement for average baseline systolic BP. The telemedicine intervention in Bove et al [12] was intense. They required patients to report their BP, heart rate, weight, steps per day, and tobacco use twice per week for the entire 6-month follow-up period. They also used an automated system that would send a message to patients when systolic BP was more than 140 mmHg, and a nurse would reach out to patients who did not send reports for 2 weeks to provide motivation. On the other hand, both Bray et al [11] and our study only involved minimal labor. In sum, the comparisons among Bove et al’s study [12], Bray et al’s study [11] and our study demonstrated that a longer length of follow-up, a larger sample, a more intensive intervention, and the requirement of participant’s systolic BP to be over 140mmHg may generate a larger effect in improving BP. However, future researchers are advised to balance the intensity of an intervention and its impact on outcomes. An intensive intervention is hard to carry out over the long term; thus, participants’ adherence to the treatment regimen can be significantly compromised. In addition, an intensive intervention also requires much more labor, which may not be economically efficient. It is recommended that future studies include participants with systolic BP over 140 mmHg to gain the most benefit of a telemedicine intervention on HTN management.

In terms of medication adherence (a secondary outcome), our study showed significant improvements in adherence at 6 months, but the Bove et al’s [12] study showed no significant change at 6 months in their general US population. This could be due to our individualized and culturally sensitive counseling sessions implemented by our bicultural and bilingual RN. During the counseling sessions, the RN discussed the importance of medication adherence and its potential challenges. The most common scenario was that participants addressed their concerns about not taking their medication daily. The RN would then advise them to associate their medication taking with ritual routine activities. For instance, for those who use incense to pray to their ancestors or God, they were advised to put the medications next to the incense. This advice was appreciated by our participants.

In terms of the other secondary outcome, self-efficacy, our study did not show improvement (mean 8.28, SD 1.25) at baseline and at 6 months (mean 7.93, SD 1.48; *P*=.74). Similarly, Fors et al’s [13] trial did not find significant improvements in self-efficacy in participants with chronic pulmonary disease or heart failure at the 6-month follow-up after their telemedicine intervention. Self-efficacy is the patient’s belief in their ability to accomplish behavior change. As such, the longer the time in between patient education and follow-up, the less likely the patient will be confident in their own abilities of self-management. In a study to determine what works and what does not work in self-management strategies for patients with chronic pain, it was found that the continuous effort to self-manage chronic pain was arduous, and motivation decreased over time [14]. Neither Fors et al’s [13] study nor our study provided intermittent, close follow-ups after the intervention. For example, in Fors et al [13], patients received 1 telephone call 1-4 weeks after their discharge date. In our intervention, patients received 4 calls every other week for 2 months. At the 6-month follow-up, it had been 4 months since the last intervention, which could explain the slight reduction in self-efficacy. Given this, future studies should investigate whether intermittent support, such as booster sessions and support groups (eg, every month), can help improve self-efficacy.

### Limitations

The limitations of this study include a small sample size, a relatively short intervention duration (6 months), and a narrow age range for participants (ranging from 51-86 years) instead of our target age range of 18 years and older. Despite these limitations, our study showed significant improvement in BP and medication adherence. However, it is anticipated that if the study had a longer duration, a larger sample size, and the requirement of systolic BP over 140 mmHg as eligibility criteria, the data could show more significant improvement in both primary (BP control) and secondary outcomes (medication adherence and self-efficacy). Another limitation is that our secondary outcomes, including medication adherence and self-efficacy, were self-reported. Thus, there may be self-report bias. In the future, other objective measures, such as pill counts, may be used to verify the data. Given our narrow age range of 51-85 years, the results of our pilot study should be carefully interpreted and applied to a future larger-scale study. For instance, since our sample did not include any adults aged 18-50 years (younger adults), the efficacy of the proposed WeChat-based intervention in medication adherence may be different from that in adults aged 51 years and older. Younger adults have their life priority in working extensively to make earnings and taking care of young children; thus, their medication adherence may be more compromised. Therefore, it is advised that for future studies, a study design and implementation be carefully discussed with experts who are very familiar with studying and taking care of younger adult patients (eg, 18-50 years).

### Conclusions

The WeChat-based HTN management program found a significant improvement in BP and medication adherence. Compared to a traditional health care model (eg, a team of a doctor, a nurse, a medical assistant, and a receptionist), the proposed WeChat-based HTN management program simply requires a 20-minute video viewing by patients on their own and another 10-20 minutes counseling with a nurse for an initial visit. For subsequent follow-up visits, in most cases, only a 5-minute phone or video chat was necessary. Thus, the intervention is of low cost and easy to implement. Further investigation is recommended to generate more robust results with a larger sample size and a longer follow-up period. In addition, the intervention should be culturally sensitive to the study population to generate clinically meaningful results. Furthermore, patients with a higher reading of BP (eg, systolic BP≥140 mmHg) should be given higher priority for HTN management to prevent further serious complications, such as a stroke.

### Implications for Clinical Practice

This social media–based program is a low-cost and easy-to-establish intervention that can be further tested to establish more robust findings, which in turn can help with HTN management in Chinese immigrants. Due to the high rates of HTN in Chinese immigrants in the United States, this intervention could be effective controling BP. In addition, because of its easy-to-establish nature, this intervention can potentially be applicable to other chronic diseases that share similarities with HTN, such as diabetes. However, it is important to note that the interventions should be managed by researchers and practitioners who are familiar with the cultural and linguistic backgrounds of the target population to generate the best outcomes for the patients through culturally sensitive care. Furthermore, this intervention should be further tested across different regions and clinical settings to establish more robust results. The long-term goal is that the intervention is implemented in clinical settings to help Chinese immigrants at large achieve better HTN management.

## Abbreviations

BP: blood pressure
CALM: Chinese Medicine as Longevity Modality
HTN: hypertension
RN: registered nurse
